# The game of language learning and rewiring biocognitive receptors

**DOI:** 10.1016/j.mex.2024.103143

**Published:** 2024-12-30

**Authors:** Sri Takshara K, Bhuvaneswari G, T.S. Pradeep Kumar

**Affiliations:** a School of Social Sciences and Languages, Vellore Institute of Technology, Vandalur - Kelambakkam Road, Chennai, 600 127 Tamil Nadu, India; b School of Computer Science and Engineering, Vellore Institute of Technology, Vandalur - Kelambakkam Road, Chennai, 600 127 Tamil Nadu, India

**Keywords:** Game based language teaching, Neuroplasticity, Neurochemicals, Epigenetics, Visual Impairment, AI, Game Based Holistic Language Teaching

## Abstract

This study introduces a framework that integrates AI-driven Game-Based Language Teaching (GBLT) with advanced neuroscience to transform language education for visually impaired learners. Built on the principles of neuroplasticity and epigenetics, the approach leverages educational psychology with the help of adaptive AI to deliver personalized, gamified learning experiences that reshape neural pathways, improve memory retention, and strengthen emotional resilience. By fostering low-stress, immersive environments, it triggers positive epigenetic changes, enhancing long-term cognitive flexibility. This method article presents an interdisciplinary inclusive scientific framework that unites gamification, technology, epigenetics, and neuroscience, empowering learners and promoting sustainable well-being, while laying the groundwork for intergenerational academic and personal growth.

• To propose a teaching methodology that aims to bridge gaps in traditional and game based language teaching by integrating neuroscience and epigenetics, creating an inclusive framework tailored to meet the unique needs of visually impaired learners.
• The method emphasizes creating stress-free, rewarding educational environments that induce neural and epigenetic changes. These changes optimize gene expression to improve retention, emotional resilience, and cognitive flexibility, ensuring both immediate academic success and lifelong developmental benefits for diverse learners.
• This framework promotes cognitive, emotional, social and biological development alongside language acquisition giving a holistic mode of growth.

## Graphical abstract

**Figure:**
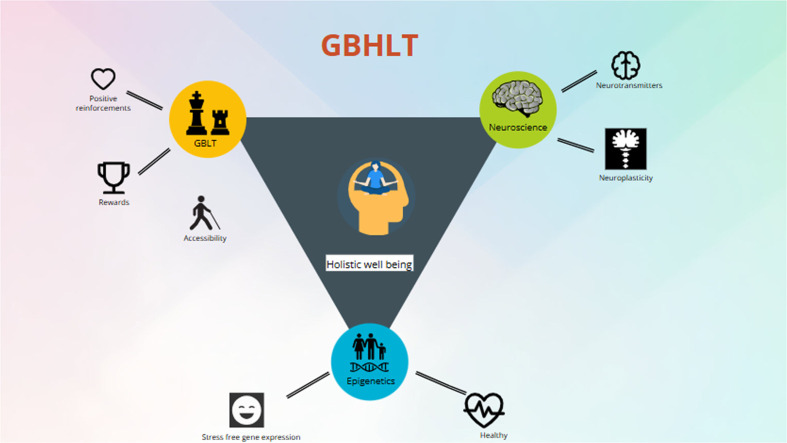

Specifications table

**Table:** 

Subject area:	Psychology
More specific subject area:	Educational Psychology, Cognitive Neuroscience, Language learning
Name of your method:	Game Based Holistic Language Teaching
Name and reference of original method:	Game Based Language Teaching by James Paul Gee
Resource availability:	N/A

## Background

Technology plays a vital role in education by continually evolving and simplifying the learning process for all learners. Among these advancements, Game-Based Language Teaching (GBLT) emerges as a technology-driven teaching method with significant benefits, particularly for visually impaired students learning a language [1]. Neural networks play a critical role in this learning process, contributing to overall well-being. ​Building on this foundation, this article proposes a method that integrates AI-powered GBLT with neuroscience principles, aiming to influence epigenetic processes and promote long-term holistic well-being.

## Epigenetics

Epigenetics, as an evolving field, has transformed our understanding of genetics by highlighting that human nature is not entirely predetermined by inherited genes; rather, it can be influenced and modified by the experiences individuals encounter, particularly during childhood making us the individuals who are made of nature and nurture [2]. This avenue of research reveals that epigenetic changes arise from the exposure to environmental factors and are especially pronounced in children, rendering them more receptive to positive instructional methods. Modifications in gene expression, influenced by environmental stimuli rather than alterations in the DNA sequence itself, underscore the significance of nurturing contexts in promoting learning [3]. In educational settings, facilitating low-stress and supportive environments can lead to beneficial epigenetic changes that enhance cognitive resilience and emotional regulation, which are critical for effective learning especially in young minds. For instance, educational games for visually impaired people designed to minimize stress, anxiety and incorporate positive reinforcement and inclusion create a rewarding atmosphere. These engaging experiences can activate epigenetic mechanisms that boost adaptability and improve long-term memory retention.

## Neuroscience: Neuroplasticity and neurotransmitters

Neuroplasticity reflects the brain's remarkable capability to reorganize itself by forming new neural connections in response to learning experiences irrespective of vision loss. In language acquisition, the process of repeated practice reinforces the neural pathways associated with speech processing, comprehension, and memory (Allen, 2019; [4]). Neurotransmitters are pivotal in attention, regulating motivation, memory retention, and emotional states [5]. AI-powered games with positive reinforcements and rewards can create immersive environments for visually impaired people that stimulate the release of these neurotransmitters, thereby enhancing cognitive flexibility and overall learning efficiency [6].

## Game-Based Language Teaching (GBLT) and the role of AI

**Fig. 1:**
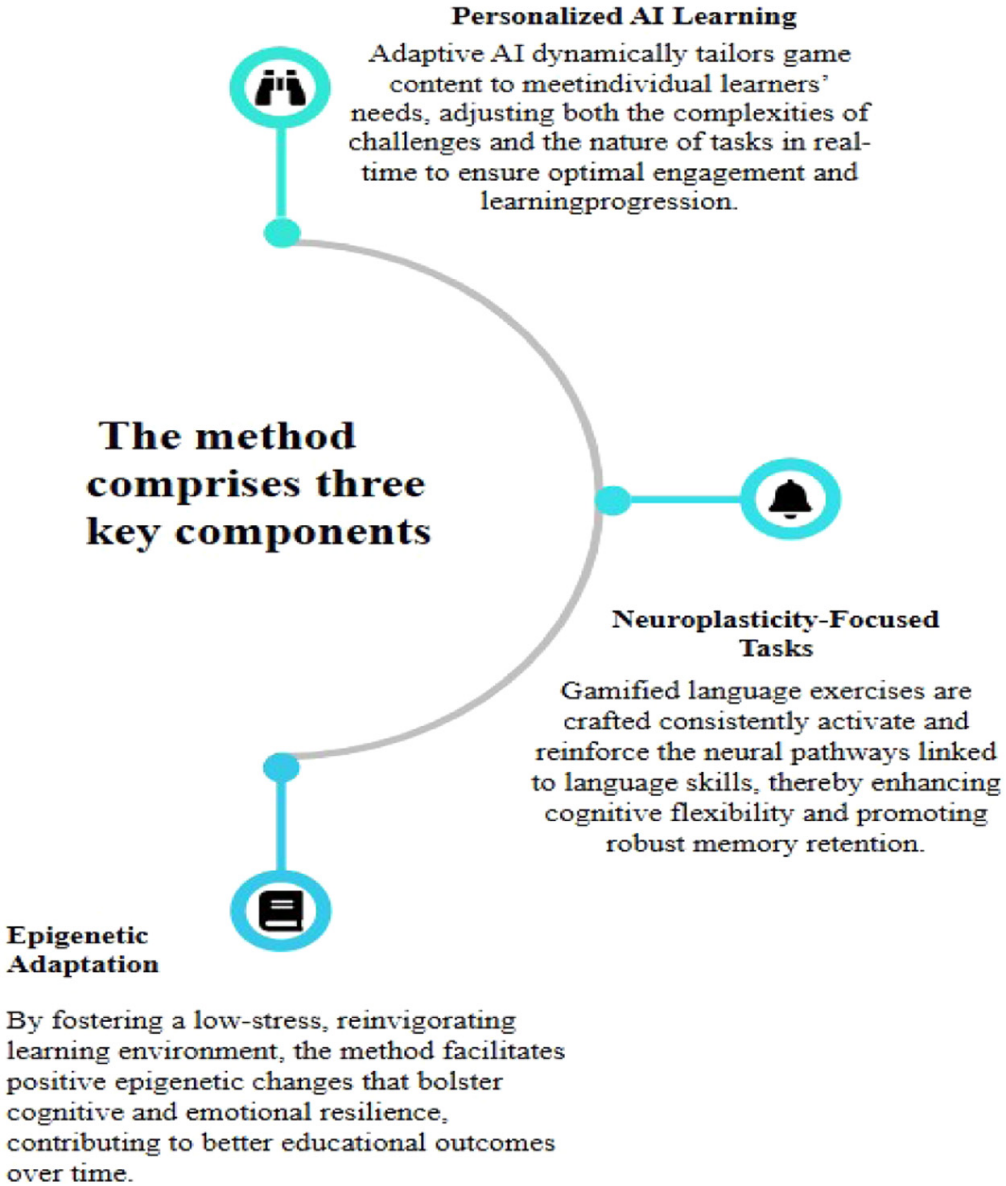
Components of game-based holistic language teaching (GBHLT).

Game-Based Language Teaching (GBLT) significantly enhances language learning by integrating interactive gameplay that promotes active learning and motivation for visually impaired children ([7]; National Center on Deaf blindness). This approach not only makes language tasks more engaging but also leads to better retention of material through a playful context [1]. The incorporation of Artificial Intelligence (AI) in GBLT further personalizes the learning experience, adapting lessons to match individual learners’ paces and skill levels while dynamically adjusting the challenges presented [8]. This synergy between GBLT and AI amplifies cognitive engagement, creating tailored tasks that foster deeper language acquisition by keeping learners motivated and focused on their objectives [9].The proposed method named Game Based Holistic Language Teaching (GBHLT) integrates AI-driven GBLT with insights from neuroscience and epigenetics, forming a comprehensive framework for language learning which can be used for all learners, particularly aimed at visually impaired students for overall development (Fig. 1).

## Significance of the method

The Game Based Holistic Language Teaching (GBHLT) stands distinct from traditional pedagogical approaches by combining AI-driven gamification, neuroscience, and epigenetics for visually impaired students into a unified framework. Unlike conventional methods, it adapts to each learner's unique needs, enhancing cognitive flexibility, emotional regulation, and long-term retention through multisensory experiences. By targeting neural pathways and inducing positive epigenetic changes, GBHLT ensures not only academic success but also emotional and psychological growth. This integrated interdisciplinary approach makes it more inclusive and effective, particularly for learners with disabilities, offering personalized and holistic learning experiences than the traditional methods.

## Method details

**Fig. 2:**
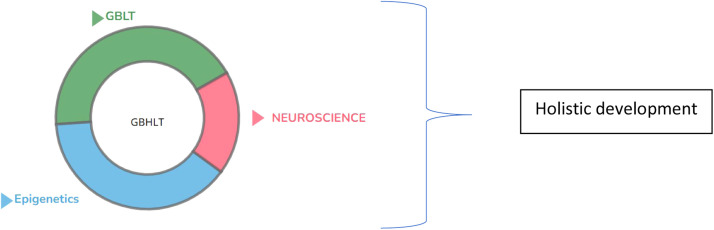
Holistic framework of GBHLT.

Game-Based Holistic Language Teaching (GBHLT) is a novel educational approach that integrates Game-Based Language Teaching (GBLT) with neuroscience and epigenetics to create a holistic, dynamic approach to language acquisition. This method builds on traditional gamification by incorporating the principles of neuroplasticity, the brain's ability to reorganize itself, and epigenetics, which highlights how environmental factors can influence gene expression. These two biological foundations ensure that learners not only engage with the lessons but also experience long-term cognitive and emotional growth (Fig. 2).

The integration of neuroscience into GBLT focuses on enhancing neuro connectivity stimulating the brain to form new neural connections through consistent, meaningful tasks. In this method, language exercises within the game are designed to activate the neural circuits responsible for language comprehension, production, and memory retention.

*For example*, repetitive tasks like vocabulary practice or sentence construction are presented through game-based challenges, encouraging the brain to strengthen and reinforce these language pathways. Over time, these exercises not only improve language fluency but also enhance cognitive flexibility, the brain's ability to adapt to new information.

Neurochemicals and neurotransmitters are chemical messengers and groups responsible for transmitting signals between nerve cells, muscles, and glands. They are fundamental to human cognition and behavior, playing a vital role in various bodily functions. They influence mood, motivation, appetite, sleep, pain perception, and numerous other physiological processes. These are triggered by the reward mechanisms within the games leading to the reduction of cortisol levels, which help regulate mood and motivation providing positive reinforcements. This neurological reinforcement ensures that learners remain engaged and motivated while improving their memory and retention of language skills. Neuroplasticity, therefore, becomes an essential component of the method, ensuring that the learning experience directly influences brain activity, making it more efficient and lasting.

Some of the major neurotransmitters are listed below, highlighting their roles in the human brain and their influence on learning and behavior. It is viewed through the lens of educational psychology, and their analysis in the context of game-based language teaching (Table 1).

**Table 1:** Major neurotransmitters and their roles in learning.

Neurotransmitter	Role	Benefits	Educational perspective	Method Implementation
**Dopamine**	Regulates motivation, reward, learning, and focus	Enhances mood, motivation, and learning capacity	Supports attention, reward systems, and motivation	Game-based elements provide rewards and challenges, increasing dopamine. Engaging AI-driven tasks lead to rewarding experiences.
**Serotonin**	Affects mood, sleep, appetite, and learning	Improves mood stability, reduces anxiety	Creates a calm and stable learning environment	Games can induce a sense of achievement, reducing stress and promoting serotonin production.
**Acetylcholine**	Involved in memory, learning, and attention	Enhances focus, attention, and memory	Crucial for memory retention and focus in education	AI driven game-based methods require high engagement and focus, stimulating acetylcholine activity.
**Endorphins**	Pain relief, stress reduction, and positive feelings	Reduces stress, improves mood, and boosts energy	Encourages a relaxed yet productive learning environment	The fun, rewarding, and immersive nature of this method increases endorphin levels, promoting positive emotions and reducing stress.
**Glutamate**	Primary excitatory neurotransmitter for learning and memory	Supports cognitive function and learning processes	Vital for memory formation and retention	The challenge and interactivity in games help enhance memory and cognitive skills through glutamate regulation.
**GABA (Gamma-Aminobutyric Acid)**	Inhibitory neurotransmitter that calms neural activity	Reduces anxiety and promotes relaxation	Reduces distractions and anxiety, enhancing learning	Games can have calming effects on students, reducing stress and increasing focus by promoting GABA production.
**Norepinephrine**	Regulates alertness, arousal, and stress response	Increases alertness, improves focus	Enhances attention and responsiveness in learning	The method's dynamic and stimulating tasks enhance focus, helping regulate norepinephrine levels.
**Oxytocin**	Promotes bonding, trust, and emotional connection	Reduces stress, enhances social bonding	Fosters trust, engagement, and collaboration	Interactive and socially engaging games foster trust and collaboration, boosting oxytocin.
**Endocannabinoids**	Regulates mood, memory, and appetite	Reduces stress, improves mood, and increases pleasure	Improves emotional stability and learning efficacy	The enjoyable and rewarding nature of games can enhance mood and motivation, promoting endocannabinoid release.

Epigenetics involves changes in gene expression due to environmental factors such as stress, reward, and engagement. It plays a pivotal role in shaping the emotional and cognitive outcomes of learners in GBHLT. Stress is a significant factor that influences gene expression by affecting various molecular processes like reduction of DNA methylation processes, potentially leading to the suppression of harmful gene activation. This reduction in stress-induced genetic changes can lower the risk of genetic diseases, anxiety related disorders and hormone-related cancers and hormone related vision loss by preventing the positive DNA patterns that contribute to these conditions. By creating a low-stress, immersive learning environment, GBHLT fosters a positive epigenetic response breaking negative psychological barriers. This environment encourages learners to engage deeply with language tasks, which influences gene expression related to memory, emotional resilience, and cognitive function.

### For example

**Fig. 3:**
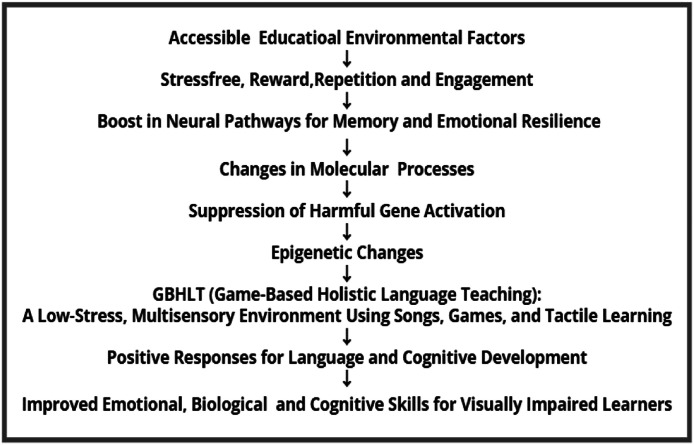
The sequential flow of holistic learning.

Game-based tasks that offer immediate rewards or positive comments make learners experience positive reinforcement that reduces stress and promotes dopamine release. This positive emotional engagement can alter epigenetic markers related to stress response, allowing learners to approach challenges with greater emotional resilience. Over time, these epigenetic changes can help enhance memory consolidation and cognitive adaptability, supporting the long-term retention of language skills (Fig. 3).

## Core tenets

At the core of GBHLT is gamification, which combines learning with interactive play. The game-based environment provides instant feedback, rewards, and challenges that keep learners motivated. Games are designed to be adaptive, adjusting the difficulty of language tasks based on the learner's progress. This personalization ensures that students remain in their Zone of Proximal Development, where tasks are neither too easy nor too difficult, promoting optimal learning conditions. Additionally, music and songs can be incorporated for better learning experiences as these are proven to enhance the functionality of neurons. Multiplayer or cooperative games that are contextual are used to foster social interaction and communication skills, allowing learners to practice language in realistic contexts. These social dynamics further strengthen the emotional and cognitive aspects of language learning, as learners with visual impairment are more likely to retain information when engaged in meaningful, collaborative experiences.

The game should be accessible, catering to all learners, including those with disabilities. AI integration, utilizing deep learning and natural language processing (NLP), will enable the game to adapt to each learner's unique needs, enhancing their learning experience. Customizing the game based on Bloom's Taxonomy ensures it addresses varying cognitive levels, offering both single-player modes for practice and multiplayer modes for fostering social interaction, inclusion, and collaboration. It should include real-life contexts or academic lessons and offer multisensory responses, catering to different levels of visual impairment. For learners with partial vision, the game could allow them to explore the screen, while blind learners can rely on auditory and haptic feedback (vibration). The AI must read and adapt to learners’ progress, facilitating personalized learning paths and offering dynamic adjustments. To enhance engagement, the game should include motivational rewards like badges, provided intermittently based on correct answers, with a dashboard for tracking progress. When the learner answers incorrectly, feedback should be constructive and positive, creating a placebo effect that fosters motivation and reduces frustration. This approach promotes self-efficacy and confidence in learners, ensuring that errors are viewed as learning opportunities rather than setbacks. Affordability is crucial for broad accessibility, so the game should be cost-effective compared to proprietary paid alternatives. Though the game can be technology-driven, combining self-paced practice and assessments with teacher guidance ensures that learners receive personalized, in-person instruction, further supporting their learning process. Incorporating advanced AI algorithms like reinforcement learning and adaptive feedback systems will allow the game to optimize learning pathways based on individual responses. This approach guarantees that all students, regardless of their disabilities or learning preferences, can engage in an enriching educational experience, fostering not just academic growth but also cognitive, emotional, and social development.

Based on these tenets, the game can be customized in terms of needs of the learners and objectives of the lessons.

The GBHLT works basically in terms of the following Equation

  Game Engagement
    ↓
  Neural Pathway Strengthening
    ↓
  Neurotransmitter Regulation
    ↓
  Epigenetic Adaptation
    ↓
  Long term learning
    ↓
  Holistic well-being

**Fig. 4:**
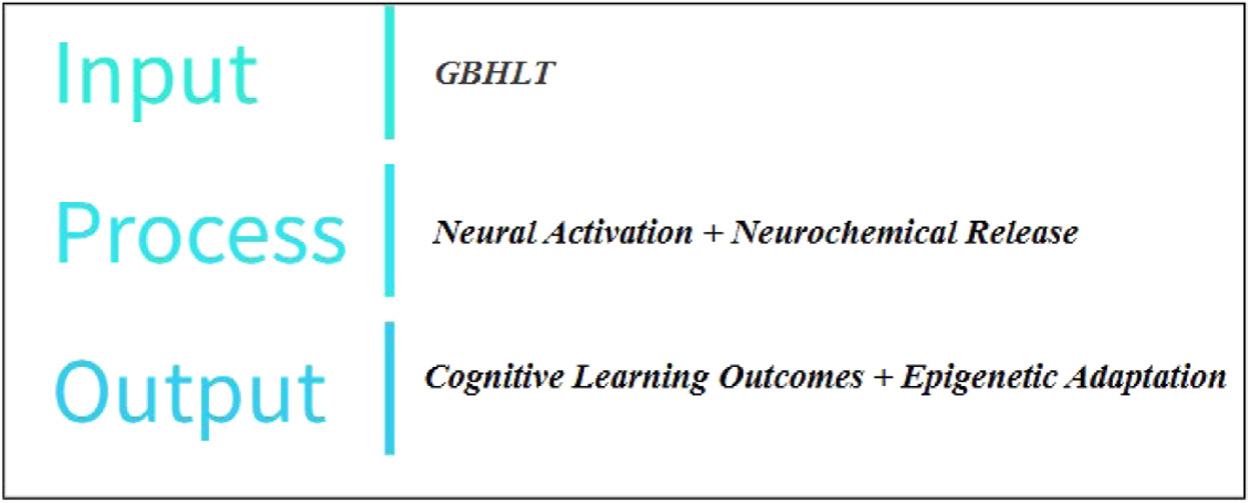
Input process output model of GBHLT.

In IPO terms (Fig. 4)

## Examples of games that can be used in GBHLT

• Audio Vocabulary Quest (Single-player)
**Context:** Learners explore an auditory world, associating words with sounds and descriptions.
**Neural Pathways:** Activates the auditory cortex, reinforcing memory pathways for language.
**Epigenetics:** Positive reinforcement through rewards induces dopamine release, fostering memory consolidation and emotional resilience in gene structures.
**Flow:** Input (Audio cue + task challenge) → Output (Word recognition and recall) → Neural activation → Positive reinforcement → Epigenetic response + Memory retention
• Collaborative Story building Adventure (Multiplayer)
**Context:** Students collaborate to create stories based on audio prompts, practicing speaking and listening.
**Neural Pathways:** Engages Broca's and Wernicke's areas for language production and comprehension.
**Epigenetics:** Positive interaction reduces anxiety, triggering the release of serotonin and improving emotional regulation.
**Flow:** Input (Audio prompts + interaction) → Output (Social communication and language fluency) → Social engagement → Neurotransmitter release → Epigenetic effect (Stress reduction)
• Memory Match with Audio Feedback (Single-player)
**Context:** Learners match audio prompts (words, sounds) to strengthen memory recall.
**Neural Pathways:** Reinforces hippocampal memory circuits.
Epigenetics: Engaging in low-stress tasks helps maintain cognitive resilience and neurogenesis reducing the risks of stress induced diseases.
**Flow:** Input (Audio-based memory task) → Output (Improved recall) → Memory circuit activation → Low-stress engagement → Epigenetic response (Cognitive resilience)
• Interactive Grammar Challenge (Single-player)
**Context:** Audio-based grammar puzzles challenge learners to form correct sentences.
**Neural Pathways:** Stimulates language processing areas like Broca's area for sentence construction.
**Epigenetics:** Positive reinforcement for correct answers triggers positive neurochemicals production removing negative psychological barriers
**Flow:** Input (Grammar challenge) → Output (Sentence formation) → Activation of language areas → Reward feedback → Epigenetic response (Neurotransmitter release)
• Real-World Scenario Exploration (Multiplayer)
**Context:** Students interact in real-world scenarios like navigating a store or discussing weather, using language for practical purposes.
**Neural Pathways:** Engages both language production and social cognition, fostering communication skills.
Epigenetics: Collaboration in real-life contexts builds social inclusion, reducing stress and promoting genetic rewiring.
**Flow:** Input (Real-world scenario + social interaction) → Output (Enhanced communication and interaction) → Social and language processing activation → Emotional regulation → Epigenetic response (Social inclusion)

## How does it benefit visually impaired students?

**Fig. 5:**
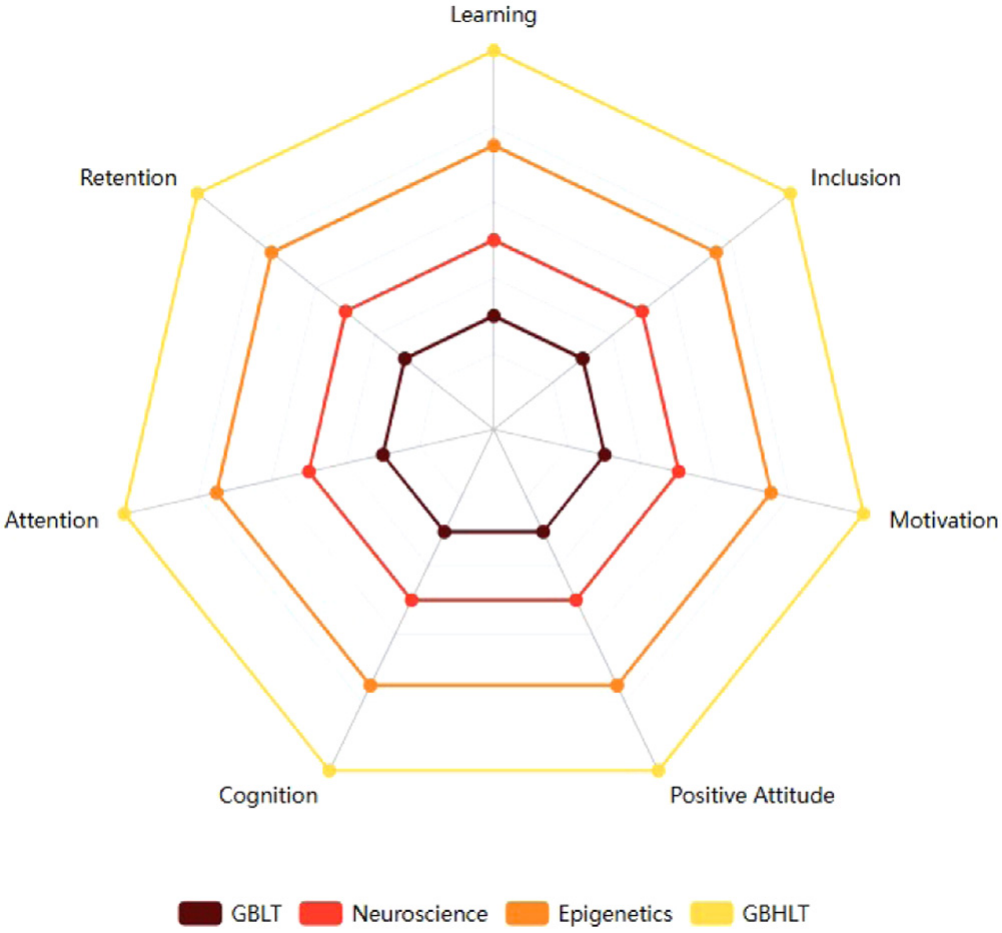
Conceptual model of GBHLT methodology.

GBHLT is distinct because it addresses the specific challenges faced by visually impaired learners. Vision loss disrupts typical sensory processing and cognitive functions like memory and attention, creating significant barriers to learning. By incorporating multisensory learning through AI-driven gamification, GBHLT engages other sensory pathways (like auditory and tactile), allowing students to compensate for their visual deficit. This approach not only enhances autonomy, promoting independent learning, but also fosters inclusion through equity while removing psychological barriers related to stress and frustration, empowering students to reach their full potential. This gives an optimistic outlook for life (Fig. 5).

## Further scope for research

• GBHLT has significant potential beyond language learning, including applications in STEM education, therapy, and other areas.
• The framework focuses on pedagogy, research on andragogy and heutagogy can be done.
• The method can be adapted for a wide range of disabilities, ensuring broader accessibility. New taxonomies can be created for game-based learning, expanding its scope across various educational settings. Long-term studies are essential to further understand its effects.
• The long-term impact of combining neuroscience, gamification, and epigenetics on language learning can be analysed.
• As epigenetics advances, GBHLT could offer valuable insights in educational psychology, particularly in analyzing brain networks and enhancing cognitive processes.

## Implications for practitioners, app developers, and academicians

• Teachers can be better equipped for technology-driven education, while app developers should prioritize inclusivity to meet diverse user needs.
• Collaboration among educators, neuroscientists, and developers can optimize the method's adaptability and effectiveness across various learning environments.

## Conclusion

The integration of neuroscience and epigenetics into game-based language teaching sets GBHLT apart from traditional methods. While gamification alone can enhance engagement, combining it with insights from neuroscience and epigenetics ensures that learners are not just passively receiving information. Instead, they are actively rewiring their brains and altering their genetic expression to better retain and process language. This method does not only focus on the immediate acquisition of language skills but also fosters long-term cognitive growth, emotional resilience, and social adaptability skills that extend beyond the classroom and enhance overall well-being. In summary, GBHLT offers a holistic, scientifically grounded approach to language learning that maximizes engagement, cognitive flexibility, and emotional resilience. By seamlessly integrating AI-driven gamification, neuroscience, and epigenetics, it creates a transformative learning experience that adapts to each learner's needs and promotes lasting growth.

## Limitations

The implementation of AI-driven gamification may require advanced technology, which may not be accessible to all students due to resource limitations.

## Ethics statements

No data involved

## CRediT authorship contribution statement

**Sri Takshara K:** Methodology, Conceptualization, Writing – original draft. **Bhuvaneswari G:** Writing – review & editing. **T.S. Pradeep Kumar:** Writing – review & editing.

## Declaration of competing interest

The authors declare that they have no known competing financial interests or personal relationships that could have appeared to influence the work reported in this paper.

## Data Availability

No data was used for the research described in the article.
